# Evaluating Mobile Information Apps for Parents of Preterm Infants After Hospital Discharge: Systematic App Review

**DOI:** 10.2196/67085

**Published:** 2026-01-19

**Authors:** Martine Jeukens-Visser, Monique Flierman, Eline Möller, Renate Giezeman, Raoul Engelbert, Daniël Bossen

**Affiliations:** 1 Department of Rehabilitation Medicine University of Amsterdam Amsterdam UMC Amsterdam The Netherlands; 2 Amsterdam Reproduction and Development Amsterdam The Netherlands; 3 Faculty of Health, Sport and Physical Activity Amsterdam University of Applied Sciences Amsterdam The Netherlands

**Keywords:** preterm, mHealth, mobile information applications, review, infant, infancy, neonate, neonatal, newborn, premature, health information, education, information quality, mobile health, app

## Abstract

**Background:**

After hospital discharge, parents of preterm infants need accessible and reliable information to gain confidence and skills in their child-caring abilities and parental autonomy. Parental need for information after hospital discharge includes topics related to prematurity, such as crying, feeding, sleeping, infant care, general health, and neuromotor development. However, parents report difficulty in finding and understanding this information. Mobile apps have the potential to improve information provision.

**Objective:**

The aim of this systematic app review was to (1) identify mobile apps for parents of preterm infants targeting the period after hospital discharge and (2) evaluate the content, quality of the app, and understandability and actionability of the information material.

**Methods:**

We systematically searched for apps in the Apple App Store, Google Play Store, and Google, along with a literature search using PubMed. Multiple keywords were used (eg, “preterm baby,” “app,” and “home”). Apps were included when they provided information for parents on topics and content related to prematurity after hospital discharge. To examine app content related to the postdischarge period, apps were reviewed, and topics were identified. The Mobile App Rating Scale (MARS) was used to measure the app’s quality, and the Patient Education Materials Assessment Tool for Audiovisual Materials (PEMAT-AV) was used to measure the understandability and actionability of the information material.

**Results:**

After the initial search, the titles and descriptions of 196 apps were screened for eligibility. Eventually, 9 English apps were included in the review. Information related to the postdischarge period constituted only a small part of the app’s content. Most commonly addressed topics related to the period at home were vaccinations, follow-up, feeding, and using home oxygen. Using the MARS, only one of the 9 apps received a good score for overall quality (“MyPreemie app”; Graham’s Foundation), and 7 apps received an acceptable score. Only 4 apps scored high on understandability of the PEMAT-AV, and 6 apps scored high on actionability. No Dutch apps were identified.

**Conclusions:**

The current availability of mobile information apps for parents of preterm infants targeting the period after hospital discharge is limited. A total of 9 English apps were identified, which contained a small portion related to the postdischarge period. This content is not comprehensive for the postdischarge period: topics indicated as relevant by parents, such as crying in preterm infants, diaper change in preterm, or parental well-being after preterm birth, are often missing. The overall quality of the apps is only acceptable. Although the reliability of the information was close to good, the understandability of the apps was moderate. Recommendations for future app development include more relevant and understandable information related to the postdischarge period, which meets the demand of parents of preterm infants.

## Introduction

Yearly, 13.4 million babies are born preterm (PT; <37 weeks of gestation), of which 2 million infants are born very preterm (VPT; <32 weeks of gestation) [1]. VPT infants are frequently discharged from the hospital before their term date [2], and parents often feel inadequately prepared to take their vulnerable infant home [3]. Without the continuous professional support of the neonatal intensive care unit (NICU), parents lack confidence or competence in infant caregiving. Parents report practical, emotional, and financial challenges at home and the need for practical support regarding baby caregiving tasks, feeding, medication, or managing unexpected health issues [4]. The uncertainty about the health, growth, and neuromotor development of their VPT infant can heighten parental anxiety and negatively affect parents’ caregiving behavior [5]. Parental confidence and competence in caring for their VPT infant can be increased by professional support and tailored information, and is thus an important approach to improve parental and infant outcomes [6,7]. Caring for a VPT infant after hospital discharge can be more demanding for parents than caring for a typically developing infant. VPT infants show different behaviors, such as reduced activity, alertness, and responsiveness, that require specific parenting skills to interpret their baby’s cues [4]. Parents therefore require information on common topics specifically targeting prematurity, such as crying (how to comfort a preterm infant), feeding (how and when to transition to solid foods), sleeping (recognize pattern of sleep and fatigue in their baby), infant regulation (help their baby to regulate), infant care (diaper change in a very small infant), general health (when to contact a pediatrician), and neuromotor development (differences in milestones between term and preterm infants) [5,8]. This information, specific to premature infants, is, however, not available on the internet [6]. Parents appreciate that general parenting websites provide accessible information on newborn topics such as feeding and digestion, but the content is perceived as less appropriate for parents with a VPT infant [6]. Therefore, practical and tailored information is necessary to increase knowledge and skills to support parents to feel confident in taking care of their preterm baby at home. To accommodate their underlying emotional needs, parents prefer information that is strength-based and confirming or reassuring in their caregiving [8].

Almost all parents in the NICU use their smartphones to search for information regarding prematurity on the internet [9]. For instance, in the Netherlands, information is provided by the Dutch parent organization (Care4neo) [10]. Facilitated by the ease of use, the 24/7 availability, and the ability to make information attractive, mobile health (mHealth) apps are promising tools to provide health information [11]. In general, parental knowledge about infant development is associated with better caregiving behavior and improved infant development [12]. Mobile apps have the potential to improve parental well-being and parenting in the perinatal period [13]. mHealth apps vary in quality, but many are of moderate quality or out of date [11,14]. A previous review on information apps targeting parents with an infant who was still admitted to the NICU showed that only a quarter of the apps for parents were considered of good quality [15]. For optimal support, parental needs for information should be incorporated in the content of the app [16]. Parents have ongoing information needs, but what they want to know changes over time [8]. After discharge home, when hospital staff support is lacking, parents need different information to feel competent in their caregiving than during their initial hospital stay.

For health care professionals and parents, it is important to be able to use high-quality apps, include engagement, functionality, aesthetics, and information quality. Therefore, the information content of mHealth applications needs to be understandable for all parents, irrespective of health literacy levels. Since preterm birth has been consistently associated with lower socioeconomic status [17], low health literacy is also a prevalent issue in parents of VPT infants. Health literacy refers to the skills needed to function effectively in the health care environment [18], and low health literacy is associated with poorer use of health care services and poorer health outcomes [18]. Parents with lower health literacy may encounter difficulties in obtaining, processing, using, and interpreting information in mHealth applications [19]. To benefit from mHealth, parents require digital health literacy skills, such as using digital devices, searching for and understanding information, and evaluating the validity of the information [20]. However, to date, little is known about the quality, understandability, and actionability of available mHealth apps designed to support parents of VPT infants after hospital discharge. Therefore, the aim of this app review was (1) to identify mobile information apps for parents of preterm infants targeting the period after hospital discharge and (2) to evaluate the content, quality of the app, and understandability and actionability of the information material.

## Methods

### Study Design

This systematic review of mobile apps followed systematic review methodology adhering to PRISMA (Preferred Reporting Items for Systematic Reviews and Meta-Analyses) standards [21] (Multimedia Appendix 1) and published conduct and reporting recommendations for systematic app store reviews [22].

### Search Strategy

To ensure the identification of relevant mobile apps, a comprehensive search was conducted, using 4 different strategies. Apps were directly searched in (1) Apple App Store for iOS and (2) Google Play Store for Android. In addition, mobile apps were also searched via (3) Google and (4) PubMed. The search in the app stores and Google was performed in December 2023. The search in PubMed was performed in May 2022. In the first 3 search strategies, keywords were used both in Dutch and in English.

The different search machines implied different search strategies. In the app stores, separate key terms (in Dutch and English) were used in the search field: “preterm baby,” “preemie,” “premature,” “NICU,” and “discharge.”

For the Google internet search, the term “app” was always used and combined with terms “preterm baby,” “preemie,” “prematurity,” NICU,” “Neonatology,” and “incubator.”

The PubMed search combined terms “parent,” “mother,” “father,” or “caregiver” AND “premature birth,” “premature infant,” “preterm,” “prematurity” AND “mobile application*,” “smartphone application*,” “health app*,” “mobile app*.” No MeSH (Medical Subject Headings) terms were used for the PubMed search. The aim of the Google internet and PubMed search was to identify more apps that were subsequently retrieved from one of the App Stores.

The search in the Apple App Store and PubMed provided a certain number of apps and papers. These were all reviewed for eligibility. In the Google Play Store, the search yielded a continuous stream of apps, many of which were not relevant to our inclusion criteria. Therefore, we limited the screening to the first 50 apps that were displayed in the search results. These are typically ordered by relevance and popularity and align with how parents would conduct such a search. In the Google web search, the first 2 pages of results were reviewed to evaluate whether an app for parents of preterm infants was described. The search ended when 2 pages did not contain new hits. Limiting search results is a common practice in app and website reviews, as later results are less likely to be accessed by parents, often align less with the search criteria, and parents are unlikely to continue their search beyond a certain point [11,15,23].

### App Selection

Several inclusion and exclusion criteria were used to select the mobile apps (Table 1). The free-of-charge criterion was used because we wanted to ensure that apps were available to all parents regardless of their socioeconomic status, income, or willingness to pay for a mobile app.

**Table 1:** Inclusion and exclusion criteria for app selection.

Condition	Inclusion criteria	Exclusion criteria
Topic	VPT^a^ infants	Typical developing infants
Timing of information	Period after hospital discharge	Only during hospital stay
Language	English or Dutch	Other languages than English or Dutch
Access	No access code	Access code required
Information in the app	Directed to parents	Not directed to parents
Download	App available for download	App not available for download
Charge	App is free of charge	Paid app

^a^VPT: very preterm.

After removing duplicates between the two app stores, the Google and PubMed search, the app descriptions and features were first screened in the Apple App Store or Google Play Store by one researcher (RG) and discussed within the research team for eligibility. Apps that fulfilled the inclusion criteria were then downloaded. Two reviewers (RG and MJ-V) screened the apps for inclusion in the full app review and discussed the eligibility within the research team.

### Data Extraction and Quality Assessment

For each app, the following data was collected: name of the app, operating system, developer and its affiliation, language, target population, year of last update, and a brief description of the app. To evaluate the postdischarge hospital content, a list of topics per app was created. To evaluate the quality of the apps and the understandability and actionability of the information material, two independent reviewers (MJ-V and RG) trained themselves to use the Mobile Application Rating Scale (MARS) [24] and the Patient Education Materials Assessment Tool for Audiovisual Materials (PEMAT-A/V) [25]. Thereafter, all included apps were independently evaluated by the two reviewers, and disagreements were resolved until consensus was reached. When no consensus was reached, the research team was involved. For each instrument, a structured data retrieval form was composed, using a spreadsheet in Microsoft Excel.

### MARS

The MARS is a tool for assessing the quality of mobile health apps. The MARS consists of 4 objective scales: “engagement” (5 items: fun, interesting, customizable, interactive, and well-targeted to audience), “functionality” (4 items: app functioning, easy to learn, navigation, and gestural design), “aesthetics” (3 items: layout, graphics, and visual appeal), and “information quality” (7 items: accuracy of app description, measurable and achievable goals, quality of information, quantity of information, visual information, credibility, and evidence-based). Each item is rated on a 5-point rating scale, ranging from 1 “inadequate” to 5 “excellent.” Each item has specific descriptions for these rating anchors. Some items have the option “not applicable”. In addition, there is one scale for “subjective quality” (4 items: recommendation of the app, estimated frequency of use, willingness to pay, and overall star rating of the app). The first 3 items are rated on a 5-point scale, and the last item on a 3-point scale. The overall mean score for the 4 objective subscales is calculated, excluding the items rated as not applicable. The MARS has a high internal consistency (α=.90) and high interrater reliability (intraclass correlation coefficient [ICC]=0.79) [24]. For this study, we used the Dutch version of the MARS [25].

### PEMAT-A/V

The PEMAT-A/V is an instrument that assesses the understandability and actionability of audiovisual patient education materials [26]. The PEMAT-A/V consists of 2 scales: understandability (13 items) and actionability (4 items). Understandability is defined as the ability of people from diverse backgrounds with varying levels of health literacy to comprehend educational material and extract key messages. Actionability is defined as the ability of learners to identify what actions can be taken on the basis of educational material information. Understandability includes 19 items evaluating the content, word choice and style, number usage, organization, layout and design, and use of visual aids. Actionability contains four items and evaluates whether the material (1) identifies an action the user can take, (2) the user is directly addressed, (3) breaks down an action into manageable steps, and (4) explains how to use the charts, graphs, tables, or diagrams to take action. Items are rated with “disagree” (0 points) or “agree” (1 point). Some items have the additional option “not applicable.” The PEMAT-A/V is designed to be completed by professionals and helps them select education material that is understandable and actionable. The PEMAT-A/V items are based on other instruments and concepts for developing educational material and are reliable for raters not trained in the use of the PEMAT-A/V. The researchers read the information in the apps and considered each item from a parental perspective, specifically a parent with low health literacy skills. The researchers did have experience with developing information for people with low health literacy skills. The scores for the two scales are calculated as a percentage, ranging from 0-100. A higher score reflects more understandability or actionability. An expert panel established the face and content validity. Interrater reliability was moderate according to Cohen κ (0.50), but with a high absolute agreement of 80% and high agreement when calculated by Gwet agreement coefficient 1 (0.71). Internal consistency was strong (Cronbach α=0.76), and the average item-total correlation=0.62. Construct validation was established based on differences in actionable and poorly actionable material, as well as a strong negative correlation between grade level and both consumer-testing results and PEMAT-A/V scores [26].

### Data Analysis

Data analysis was performed using IBM Statistical Package for Social Sciences software (IBM SPSS; version 26). The MARS item scores were averaged for the engagement, functionality, aesthetics, and information subscales. These scores for app quality were then averaged, creating a mean (SD) app quality score. Descriptive statistics were used to summarize the results of the MARS and the PEMAT-A/V. To evaluate consistency between raters, the ICC between the raters was calculated for the MARS and the PEMAT-A/V. Rater agreement was examined by ICC based on a 2-way mixed-effects model. An ICC of <0.50 is considered poor, 0.51-0.75 as moderate, 0.76-0.89 as good, and >0.90 as excellent.

## Results

### Search Results

**Figure 1:**
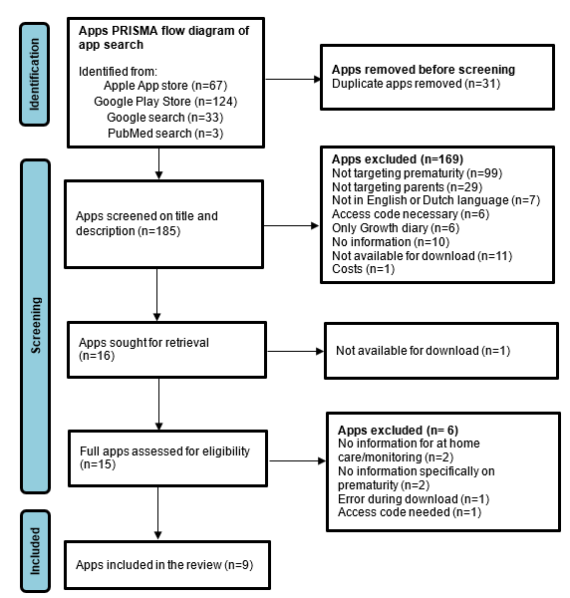
PRISMA (Preferred Reporting Items for Systematic Reviews and Meta-Analyses) flow diagram of search for apps for parents of preterm infants after hospital discharge.

The search yielded 191 apps in the Apple and Google Play stores, and additionally 36 apps in Google and PubMed. After removing duplicates, 185 apps remained (Figure 1). Based on the title and description in the app stores, 169 apps were excluded. The majority of the excluded apps did not contain information on preterm-born infants (n=99; 58%), did not target parents but health care professionals (n=29; 17%), or did not contain information, but for instance only growth diaries (n=6; 4%). Only one app was excluded because it was a paid app. A total of 12 apps (6%) that were identified via Google or PubMed could not be retrieved anymore in the app stores. The remaining 16 apps were downloaded and screened for inclusion in the full app review. One app was not available for downloading. Finally, 9 apps fulfilled the inclusion criteria and were included in the final analysis. The majority of the apps were available in both app stores (n=5), 3 apps were only available in the Apple App Store, and 1 app was only available in the Google Play Store. In addition, 2 apps were also described in a scientific paper. One paper describes the content of the MyPreemie app, based on an earlier book, Preemies: the Essential Guide for Parents of Premature Babies, supplemented with new tools [27]. The co-design approach of the Preterm Connect app has been described across 3 settings with different social, economic, and cultural participants [28]. The preliminary findings show similar parental needs, but different preferences across the study populations.

### Characteristics of the Apps

The apps were developed in the United States of America (n=4; A2, A4, A7, and A9), the United Kingdom (n=2; A3 and A5), Australia (n=1; A6) and New Zealand (n=1; A1), and South Africa (n=1; A8; Table 2). All 9 apps were in English; no Dutch apps fulfilled the inclusion criteria. One app (A2) had information in 25 languages, and another app (A4) was also available in Spanish. The apps were developed by reputable sources, including hospitals (n=4; A1, A3, A6, and A7), nongovernmental organizations (n=3; A2, A4, and A5), and universities (n=2; A8 and A9). The last update of the app varied between 3 weeks and 3 years, with the majority of the apps updated more than a year ago. The size of the apps varied between 7.2 and 102.2 MB. In addition, 3 apps were downloaded more than 1000 times (A1, A2, and A4) and received positive ratings ranging from 4.0 to 4.9 on a scale from 0-5.

**Table 2:** General characteristics of the apps for parents of preterm infants after hospital discharge.

ID	Name	Operating system	Country	Version	Last update	Target population	Developer	Affiliation	Brief description	Languages	Size (Mb)	Rating	Downloads
A1	Babble NZ Neonatal Family App	Apple iOS; Android	New Zealand	2.7.1	2021	Parent with a baby in the NICU^a^.	Neonatal unit at Midcentral Health	Hospital	A reliable source of information about the NICU.	English	7.2	4.9 (n=10)^b^; N/A^c,e^	1000+
A2	Birth & Beyond	Apple iOS; Android	The United States	1.16	2022	Mothers of newborn babies.	Global Health Media Project	NGO^d^	48 videos in 30 languages.	25 languages	10.7	4.8 (n=26)^b^; N/A^e^	10,000+
A3	Family Delivered Neonatal Care (IFDC)	Apple iOS	The United Kingdom	1.1.11	2023	Parents in the NICU.	Imperial College healthcare NHS trust	Hospital	The app offers up-to-date and comprehensive educational material, a developmental timeline, and diary functions to document the neonatal journey.	English	29.2	N/A^e^	N/A
A4	MyPreemie app	Apple iOS; Android	The United States	2.3.1	2020	Families of premature babies.	Graham’s Foundation	NGO	Toolkit for the practical and emotional needs of families of premature babies.	English; Spanish	35.4	4.0 (n=182)^b^; 5 (n=1)^e^	10,000+
A5	My Prem Baby	Apple iOS	The United Kingdom	1.8.1	2023	Parents of a premature baby.	Tommy’s	NGO	Track and monitor the journey with your premature baby.	English	50.9	N/A^e^	N/A
A6	Neonatal Care and Me	Apple iOS; Android	Australia	N/A	2021	Parents of a baby in the NICU, the special care nursery of pediatrics.	South Western Sydney Local Health District	Hospital	Tools while the baby is in the hospital and beyond, and while being guided by a health professional.	English	102.2	N/A^b^; N/A^e^	N/A
A7	Our Journey in the NICU	Apple iOS	The United States	2.1	2020	Families of children in the NICU.	Phoenix Children’s Hospital	Hospital	Identify what families need to know before taking their child home from the hospital.	English	8.2	N/A^e^	N/A
A8	Preemie Mom Care	Android	South Africa	2.0.5	2020	Mothers of hospitalized premature infants.	UCT Human Computer Interaction lab	University	Provides supportive information to mothers of hospitalized premature infants as they partake in the care of their infant.	English; Afrikaans; Xhosa	24.8	N/A^b^	500+
A9	PretermConnect	Apple iOS; Android	The United States	1.8.5	2023	Parents of a preterm baby.	Chih H Wang	University	Connect with other women through forms for preterm birth. Articles and videos about caring for a preterm baby and yourself.	English	63.5	N/A^b^; N/A^e^	50+

^a^NICU: neonatal intensive care unit.

^b^Rating in Google Play.

^c^N/A: not available.

^d^NGO: nongovernmental organization.

^e^Rating in Apple App Store (range 0-5).

### App Content

Most of the information in all apps was directed to the period in the NICU. The quantity of information for the posthospital discharge period was limited. Some apps have one “chapter” that covers the postdischarge period (A5, “at home with baby;” A9, “Parenting at home”), whereas other apps have subthemes within a chapter (A4, “Preemie Parenting” and “going home”). Topics that were addressed varied widely between the apps (Table 3). The most common topics that were covered in the apps related to the period post discharge were: vaccinations, follow-up, and recognizing signs of illness. More practical information was provided on feeding, using home oxygen, and sleep (Table 3). Less often, the apps provided information on aspects that were reported as relevant by parents [5,8] as diaper change (A5 and A9), crying (A1, A7, and A9), or parental well-being (A4, A5, and A9).

**Table 3:** Most common postdischarge topics and functionalities of the apps.

App content		Number of apps	App IDs
**Common topics post discharge**			
	Feeding	7	A2, A3, A4, A5, A6, A8, and A9
	Vaccinations	6	A1, A2, A3, A4, A5, and A9
	Follow-up	6	A1, A3, A4, A5, A8, and A9
	Signs of illness in a baby	5	A1, A2, A7, A8, and A9
	Sleep	5	A1, A3, A5, A7, and A9
	Home oxygen	4	A3, A4, A5, and A7
	Bathing	4	A5, A6, A7, and A9
**Functionalities**			
	Monitoring or tracking (diary, growth, and weight)	3	A4, A5, and A6
	Making notes or saving questions	3	A4, A5, and A7
	Sharing information	3	A2, A4, and A5
	Saving articles	3	A1, A2, and A9
	Community groups	1	A9

Besides information provision, the apps also included other functionalities, including monitoring and tracking of infants’ weight and height, amount and duration of feeding, or parental mood (A4, A5, A6, and A7; Table 3). The option of making notes was also provided by 4 apps (A3, A4, A7, and A9). Sharing information from the app with others was available in 3 apps (A2, A4, and A5). Community groups were only incorporated in a single app (A9).

### Quality of the Apps (MARS)

The interrater reliability of the MARS of the two raters was high (ICC=0.99, CI 0.98-0.99). The overall mean quality (range 0-5) of the 9 apps was 3.4 (SD 0.5; range 2.3-4.3; Table 4). The majority of the apps (n=7) scored between acceptable to good, one app (A7) scored below acceptable, and only one app (A4) scored above good. There was a difference in the ratings between the 4 objective MARS scales. Engagement was rated as poor to acceptable (mean 2.8, SD 0.6; range 1.8-3.4), specifically due to low scores on entertainment, customization, and interactivity. The aesthetics domain was acceptable (mean 3.2, SD 1.0; range 1.7-5). Information quality and functionality were close to good (mean 3.8, SD 0.6; range 2.3-4.3), and (mean 3.9, SD 0.5; range 3.3-4.8), respectively. Several apps received a good score (>4) for information quality (A2, A4, A5, A6, and A9), functionality (A2, A3, A4, and A8), aesthetics (A4 and A6), and overall mean quality (A4). None of the apps received a good score for engagement. The subjective quality (total range 0-18) ranged from 8 to 16, with a mean of 12.6 (SD 3.0). A total of 7 apps (A1, A2, A3, A4, A5, A6, and A9) received a good score for subjective quality.

### Understandability and Actionability of the Apps (PEMAT-AV)

The interrater reliability of the PEMAT-AV between the two raters was high (understandability ICC=0.89, 95% CI 0.55-0.98; actionability ICC=0.91, 95% CI 0.59-0.98). The mean understandability of the apps was 78% (SD 12%), ranging from 55% to 100% (Table 4). Only a single app (A2) scored the maximum of 100% for understandability. Lower ratings were obtained when lacking a summary of the information or visual cues to draw attention to key points. The mean actionability was 85% (24%; range 33% to 100%). Lower ratings were obtained when not addressing the user directly or not breaking the action down into manageable, explicit steps. A total of 6 apps (A2, A3, A5, A6, A7, and A9) received the maximum score of 100% for actionability.

**Table 4:** Quality of the apps and the understandability and actionability of the information material.

ID	Name	MARS^a^								PEMAT A/V^b^	
		A^c^	B^d^	C^e^	D^f^		Mean^g^ (SD)	E^h^	U^i^		AC^j^
A1	Babble	3	3.5	3.7	3,8		3.5 (0.3)	14	75		33
A2	Birth & Beyond	2.2	4.8	2	4.2		3.3 (1.2)	10	100		100
A3	IFDC	3.4	4	3.3	3.5		3.6 (0.3)	14	75		100
A4	MyPreemie app	3.2	4.8	5	4.2		4.3 (0.7)	16	82		67
A5	My Prem Baby	3.2	3.8	3	4		3.5 (0.4)	14	55		100
A6	Neonatal Care	3	3.6	4	4.3		3.7 (0.5)	15	83		100
A7	Our Journey in NICU	1.8	3.3	1.7	2.3		2.3 (0.6)	8	75		100
A8	Preemie Mom Care	2.4	4	3.3	3.4		3.3 (0.6)	8	82		67
A9	PretermConnect	3.4	3.5	3	4.2		3.5 (0.4)	14	75		100
	Mean (SD)	2.8 (0.6)	3.9 (0.5)	3.2 (1.0)	3.8 (0.6)		3.4 (0.5)	12.6 (3.0)	78 (12)		85 (24)

^a^MARS: Mobile App Rating Scale.

^b^PEMAT-A/V: Patient Education Materials Assessment Tool for Audiovisual Materials evaluation.

^c^Engagement.

^d^Functionality.

^e^Aesthetics.

^f^Information quality.

^g^Overall mean quality.

^h^Subjective quality.

^i^Understandability.

^j^AC: actionability.

## Discussion

### Principal Findings

This app review provides insight into the availability, content, quality of the apps, and the understandability and actionability of the information material for parents of preterm infants after hospital discharge. A total of 9 apps were identified that provided information after hospital discharge, but the amount of information on the postdischarge period was limited in all apps. Only one app was of overall good quality, while the mean overall quality was between acceptable and good. The understandability and actionability of the apps were respectively moderate and good.

Although our inclusion criteria focused on the postdischarge period, the apps in this review contained primarily information for the NICU period. The lack of high-quality and understandable apps found in this review is in contrast with the needs of parents of VPT infants after hospital discharge. Parents of VPT infants have reported challenges when they are at home regarding the availability and usability of information [8]. For parents who struggle to seek information, finding an app with appropriate and reliable content will be even more difficult, particularly for those with low health literacy skills [29]. Health care professionals, such as nurses, pediatricians, or pediatric physical therapists, have a responsibility to support parents in their search for relevant and reliable information during their hospital stay. As parental competence was found to decrease after discharge home, it is an important strategy to improve parental confidence in taking care of their VPT infant [30]. When parents and infants are at home, without direct access to a health care professional, apps have the potential to provide health information to parents and can be accessed when and where needed.

Mobile apps can, however, not replace in-person care. Effective use of apps requires guidance from health care providers, as combining digital tools with professional support has been shown to enhance parental confidence [31]. This is even more important for parents with limited health literacy or digital literacy, who are at higher risk of misunderstanding or misapplying information [18,19]. Our findings confirmed that the understandability of many apps is limited, largely due to complex medical terminology and text-heavy formats. This can particularly exclude parents with low health literacy, widening the existing digital divide [32]. Improving understandability, for example, through audio, video, simplified language, and multilingual options, along with professional support, is essential to make apps usable and effective for all parents.

Apps that cover both the period in hospital and after discharge can be beneficial to parents by providing relevant information throughout the different phases. In a previous review of 18 apps in the NICU context [15], only 5 were included in our review, indicating that most NICU apps do not cover topics post-discharge. There was variability in the amount of postdischarge information, the topics, the emphasis within the topics, and how the information was presented. Unfortunately, the topics do not seem to correspond with the information needs of parents upon discharge [5,8], such as daily infant care, neuromotor development, as well as the impact of prematurity on parents. Instead, most topics are focused on vaccinations, follow-up, and using home oxygen.

Despite the use of Dutch search terms, no Dutch apps were retrieved in the App stores that fulfilled the inclusion and exclusion criteria. The Dutch apps that were found in the Google search were no longer available in the Google Play Store or Apple Store. The majority of the apps evaluated in this review were last updated over one year ago. This lack of updates is in line with a scoping review about problems and barriers related to the use and implementation of apps [33] and, consequently, impedes usability and user experience, which ultimately affects the effectiveness of applications. Apps without active maintenance quickly become outdated due to evolving technology, guidelines, and operating systems [34]. This underscores the necessity of a viable business model and continuous refinement and maintenance after initial development [35]. Sustainable funding for apps is essential, but there are currently few resources available. Partnerships between industry and research may offer a possible solution for some apps.

Only one app had good overall quality, whereas the mean overall quality of the apps was merely acceptable. This is in agreement with an earlier app review, where the mean overall quality was also acceptable [15]. Specifically, aspects within the domains of engagement and aesthetics could be improved. The apps scored particularly low on the engagement domain of the MARS, lower than acceptable. This subscale assesses whether the app is fun, interesting, customizable, interactive, and well-targeted to the audience. Lack of engagement is a common barrier related to the use of mHealth apps and is associated with low adherence [33]. Different functionalities can facilitate parental engagement with an app. A low rating on the Engagement domain suggests improvements are needed. Increasing engagement through entertainment appears not suitable for an app that provides information related to prematurity. However, the app could be customizable or interactive, and should certainly be well-targeted to the audience. If not, this latter aspect would certainly hinder the use of the app. A positive finding from our review was that the domain “information quality” of the apps was close to good. Reliable information is important as it may decrease parental stress and support better caregiving behavior [12]. This also matches the parental needs for reliable information and is probably a result of the reputable sources (hospitals and universities) that developed the apps. This is in contrast with two previous studies in which only 31% and 40% of the websites provided accurate and reliable information for parents of premature babies [6,11].

The understandability of the apps was scored as moderate, largely due to the primarily text-based information, indirect communication with users, and frequent use of medical terminology. In contrast, the app Birth and Beyond (Global Health Media Project) circumvented this problem by using only videos, in multiple languages. During stressful periods, such as hospital discharge, information should be presented in a clear and accessible manner, particularly for parents with low health literacy. For these individuals, the digital divide can be further exacerbated when the information is difficult to comprehend. The hospitals, universities, and nongovernmental organizations create apps with reliable information, but it may not be easily understood by all users. To meet the informational needs of all parents, apps need to be more understandable. Co-design that incorporates both health care professionals’ and parents’ perspectives can enhance app understandability by identifying the preferences and needs of the target group [34]. Reducing text, written at accessible reading age levels, using multiple languages, and incorporating audio and visual formats may improve understandability.

Only 2 papers were retrieved that described the development of an app [27,28], indicating a general lack of transparency about co-creation. None of the 9 apps have been assessed for their impact on parental outcomes. A study on the NICU2HOME app (CF Garfield) [31,36] showed that parental self-efficacy and satisfaction with care improved in parents of preterm infants. This mobile app has not been included in this review, as an access code was required. More research is needed to evaluate the use of apps, parental satisfaction, and the effects of app use on parental outcomes.

### Limitations

First, only English and Dutch apps, free and without an access code, were included in the search, thereby possibly missing potential relevant apps. Second, other online resources that provide information to parents, such as websites, were also excluded. Also, progressive web applications were not captured in our search, as these are not available in the searched app stores. Third, the search for apps is time-dependent. Some apps are only available for a short time in the app stores, and replication of the search is therefore difficult. This became clear when apps identified through Google or PubMed were not available in the app stores. During the initial screening of app descriptions and features, followed by a secondary screening for inclusion, app content has been checked to decide whether it also contained information related to the postdischarge period. Fourth, it may be possible that apps have been excluded during the initial screening because the description did not refer to information related to the postdischarge period. However, this information was then likely not substantial and would also not appeal to parents. Fifth, the assessment of the quality of the apps and the understandability and actionability of the information material has been done by the MARS and PEMAT A/V. These are validated tools used by professionals. The researchers were familiar with the parental needs for information [8] and did consider the parental perspective during the evaluation of the apps. However, direct information from parents of a preterm infant has not been taken into account. As parents are the key users, their experiences are most valuable, and their engagement is important to ensure the content meets their needs. A next step would be to include parents to evaluate their experiences with good-quality apps. Finally, although it was evident that information on the postdischarge period was limited, we did not quantify the amount of information provided in the apps. Topics on postdischarge information were identified using a checklist and compared to previously recognized parental needs for information. While the lack of information on the posthospital discharge period was apparent, no specific measurement was conducted to assess the extent of information for the hospital or home environment. Furthermore, an assessment of the relevance of the topics was also lacking. Future work may establish new methods to incorporate these aspects.

### Recommendations

During the post hospital-discharge period, parents of preterm infants need evidence-based, reliable, and practical information. Mobile apps have the potential to offer this information in an accessible way. Currently, few good quality apps exist that contain reliable and understandable information, as the My Preemie app or Preterm Connect. However, more relevant information that matches the needs of parents of VPT infants after hospital discharge is necessary. Future development of digital support tools should also consider solutions that bridge the gap between in-hospital and at-home care by extending access to apps currently limited to the NICU setting. Co-design with parents has been shown to improve the relevance and understandability of health apps [37]. We not only recommend that future apps should be developed or adapted in co-creation with end users, but also that the development process is clearly reported. Research into the use and satisfaction of the parents should establish what information is key for parents, as well as how to deliver this information. In addition, the accessibility and understandability of an app need to be evaluated among parents with a preterm-born infant. The next step would be to evaluate the effect of the information app on parental outcomes as parenting skills, knowledge, and confidence.

### Conclusion

The current availability of mobile information apps for parents of preterm infants targeting the period after hospital discharge is limited and not in line with the high parental demand. A total of 9 English apps were identified containing information on the postdischarge period. However, the apps contained limited content for the period at home. The overall quality of the apps was just acceptable, but the information quality was close to good. The understandability of the apps was moderate. Developing apps in co-creation with the end-users to better match their needs and increase the understandability is recommended.

## Data Availability

The datasets generated and analyzed during this study (ie, the MARS and PEMAT scores from two independent reviewers) are available in the Figshare repository [38]. The mobile apps assessed in this review are publicly accessible via the Apple App Store and Google Play Store.

## Appendix

Multimedia Appendix 1 PRISMA 2020 checklist.

## Abbreviations

ICC: intraclass correlation coefficient
MARS: Mobile App Rating Scale
MeSH: Medical Subject Headings
mHealth: mobile health
NICU: neonatal intensive care unit
PEMAT-AV: Patient Education Materials Assessment Tool for Audiovisual Materials
PRISMA: Preferred Reporting Items for Systematic Reviews and Meta-Analyses
PT: preterm
VPT: very preterm

## References

[1] Ohuma EO, Moller A, Bradley E, Chakwera S, Hussain-Alkhateeb L, Lewin A, Okwaraji YB, Mahanani WR, Johansson EW, Lavin T, Fernandez DE, Domínguez GG, de Costa A, Cresswell JA, Krasevec J, Lawn JE, Blencowe H, Requejo J, Moran AC (2023). National, regional, and global estimates of preterm birth in 2020, with trends from 2010: a systematic analysis. Lancet.

[2] Edwards EM, Greenberg LT, Horbar JD, Gagliardi L, Adams M, Berger A, Leitao S, Luyt K, Ehret DE, Rogowski JA (2023). Discharge age and weight for very preterm infants in six countries: 2012-2020. Neonatology.

[3] Petty J, Whiting L, Green J, Fowler C (2018). Parents' views on preparation to care for extremely premature infants at home. Nurs Child Young People.

[4] Green J, Fowler C, Petty J, Whiting L (2021). The transition home of extremely premature babies: An integrative review. J Neonatal Nurs.

[5] Davis-Strauss S, Johnson E, Lubbe W (2020). Information and support needs of parents with premature infants: an integrative review. J Early Interv.

[6] Alderdice F, Gargan P, McCall E, Franck L (2018). Online information for parents caring for their premature baby at home: a focus group study and systematic web search. Health Expect.

[7] Setiawan J, Mannix T, Sweet L (2019). Understanding the effects of neonatal early discharge on parents: a literature review. J Perinat Neonatal Nurs.

[8] Flierman M, Bossen D, de Boer R, Vriend E, van Nes F, van Kaam A, Engelbert R, Jeukens-Visser M (2024). Parents' information needs during the first year at home with their very premature born child; a qualitative study. PEC Innov.

[9] Orr T, Campbell-Yeo M, Benoit B, Hewitt B, Stinson J, McGrath P (2017). Smartphone and internet preferences of parents: information needs and desired involvement in infant care and pain management in the NICU. Adv Neonatal Care.

[10] (2025). Care4neo.

[11] Dol J, Richardson B, Boates T, Campbell-Yeo M (2019). Learning to parent from Google? Evaluation of available online health evidence for parents of preterm infants requiring neonatal intensive care. Health Informatics J.

[12] Leung CYY, Suskind DL (2020). What parents know matters: parental knowledge at birth predicts caregiving behaviors at 9 months. J Pediatr.

[13] Chua JYX, Shorey S (2022). Effectiveness of mobile application-based perinatal interventions in improving parenting outcomes: a systematic review. Midwifery.

[14] Eysenbach G, Powell J, Kuss O, Sa E (2002). Empirical studies assessing the quality of health information for consumers on the world wide web: a systematic review. JAMA.

[15] Richardson B, Dol J, Rutledge K, Monaghan J, Orovec A, Howie K, Boates T, Smit M, Campbell-Yeo M (2019). Evaluation of mobile apps targeted to parents of infants in the neonatal intensive care unit: systematic app review. JMIR Mhealth Uhealth.

[16] Spence CM, Stuyvenberg CL, Kane AE, Burnsed J, Dusing SC (2023). Parent experiences in the NICU and transition to home. Int J Environ Res Public Health.

[17] Granés L, Torà-Rocamora I, Palacio M, De la Torre L, Llupià A (2023). Maternal educational level and preterm birth: exploring inequalities in a hospital-based cohort study. PLoS One.

[18] Berkman ND, Sheridan SL, Donahue KE, Halpern DJ, Crotty K (2011). Low health literacy and health outcomes: an updated systematic review. Ann Intern Med.

[19] Enlow E, Gray M, Wallace-Keeshen S, D'Agostino J, Abbasi S, Lorch S (2019). Health literacy of parents of very preterm infants at NICU admission and discharge: a prospective cohort study. J Perinatol.

[20] Donelle L, Hiebert B, Hall J (2023). An investigation of mHealth and digital health literacy among new parents during COVID-19. Front Digit Health.

[21] Moher D, Shamseer L, Clarke M, Ghersi D, Liberati A, Petticrew M, Shekelle P, Stewart LA, PRISMA-P Group (2015). Preferred reporting items for systematic review and meta-analysis protocols (PRISMA-P) 2015 statement. Syst Rev.

[22] Grainger R, Devan H, Sangelaji B, Hay-Smith J (2020). Issues in reporting of systematic review methods in health app-focused reviews: a scoping review. Health Informatics J.

[23] Hall M, Challacombe F, Curran C, Shennan A, Story L (2023). Googling preterm prelabour rupture of the membranes: a systematic review of patient information available on the internet. BJOG.

[24] Stoyanov SR, Hides L, Kavanagh DJ, Zelenko O, Tjondronegoro D, Mani M (2015). Mobile app rating scale: a new tool for assessing the quality of health mobile apps. JMIR Mhealth Uhealth.

[25] Tency I, Van Hecke A, Coorevits P (2019). Assessing the quality of pregnancy apps through development and validation of the Dutch version of the Mobile Application Rating Scale (MARS).

[26] Shoemaker SJ, Wolf MS, Brach C (2014). Development of the patient education materials assessment tool (PEMAT): a new measure of understandability and actionability for print and audiovisual patient information. Patient Educ Couns.

[27] Doron MW, Trenti-Paroli E, Linden DW (2013). Supporting parents in the NICU: A new app from the US, ‘MyPreemie’. J Neonatal Nurs.

[28] Jani S, Nguyen A, Abraham Z, Scala M, Blumenfeld Y, Morton J, Nguyen M, Ma J, Hsing JC, Moiwa-Grant M, Profit J, Wang C J (2021). PretermConnect: Leveraging mobile technology to mitigate social disadvantage in the NICU and beyond. Semin Perinatol.

[29] Lee H, Jin S, Henning-Smith C, Lee J, Lee J (2021). Role of health literacy in health-related information-seeking behavior online: cross-sectional study. J Med Internet Res.

[30] Polizzi C, Perricone G, Morales MR, Burgio S (2021). A study of maternal competence in preterm birth condition, during the transition from hospital to home: an early intervention program's proposal. Int J Environ Res Public Health.

[31] Garfield CF, Kerrigan E, Christie R, Jackson KL, Lee YS (2022). A mobile health intervention to support parenting self-efficacy in the neonatal intensive care unit from admission to home. J Pediatr.

[32] Estacio EV, Whittle R, Protheroe J (2019). The digital divide: examining socio-demographic factors associated with health literacy, access and use of internet to seek health information. J Health Psychol.

[33] Giebel GD, Speckemeier C, Abels C, Plescher F, Börchers K, Wasem J, Blase N, Neusser S (2023). Problems and barriers related to the use of digital health applications: scoping review. J Med Internet Res.

[34] Noorbergen TJ, Adam MTP, Teubner T, Collins CE (2021). Using co-design in mobile health system development: a qualitative study with experts in co-design and mobile health system development. JMIR Mhealth Uhealth.

[35] van Gemert-Pijnen JE W C, Nijland N, van Limburg M, Ossebaard HC, Kelders SM, Eysenbach G, Seydel ER (2011). A holistic framework to improve the uptake and impact of eHealth technologies. J Med Internet Res.

[36] Garfield CF, Lee YS, Kim HN, Rutsohn J, Kahn JY, Mustanski B, Mohr DC (2016). Supporting parents of premature infants transitioning from the NICU to home: a pilot randomized control trial of a smartphone application. Internet Interv.

[37] Huang Z, Benyoucef M (2022). A systematic literature review of mobile application usability: addressing the design perspective. Univ Access Inf Soc.

[38] (2025). The MARS and PEMAT scores from two independent reviewers. Figshare.

